# A mixed-methods study of autistic adults’ healthcare independence over time

**DOI:** 10.1016/j.hctj.2023.100029

**Published:** 2023-11-07

**Authors:** Daniel Gilmore, Deondray Radford, Alex Coyne, Christopher Hanks, Daniel L. Coury, Amy Hess, Jennifer H. Garvin, Brittany N. Hand

**Affiliations:** a School of Health and Rehabilitation Sciences at The Ohio State University, 453 W 10th Ave., Columbus, OH 43210, United States; b Center for Autism Services and Transition, The Ohio State University Wexner Medical Center, 3691 Ridge Mill Dr, Hilliard, OH 43026, United States; c Nationwide Children’s Hospital, 700 Children's Dr, Columbus, OH 43205, United States

**Keywords:** Autistic adult, Healthcare independence, Mixed methods, Primary care, Survey

## Abstract

**Background:**

Healthcare independence refers to an individual’s ability to participate in and manage their healthcare by using specific skills like communicating with providers and scheduling appointments. Understanding healthcare independence among autistic young adults is important to designing healthcare systems that provide equitable support for autistic people throughout their lives.

**Objective:**

To quantify changes in autistic adults’ healthcare independence over time and understand factors associated with change in healthcare independence.

**Methods:**

We administered a measure of healthcare skills, the Transition Readiness Assessment Questionnaire (TRAQ), to n = 27 autistic young adults who provided a self-report, and n = 21 autistic young adults who participated via proxy-report by supporters, at one autism-specialized primary care clinic. Participants completed the TRAQ at baseline, six months, and 12 months. We used repeated measures generalized linear mixed models to quantify changes in healthcare independence over time, controlling for demographic factors, executive functioning, restrictive and repetitive behaviors, and number of clinic visits. To understand factors associated with change in healthcare independence, we completed follow-up semi-structured interviews with n = 6 autistic young adults and n = 5 supporters of autistic young adults.

**Results:**

Autistic young adults who participated via self-report showed statistically significantly increases in healthcare independence between baseline and 12 months and between six months and 12 months, and significant increases on most TRAQ subdomains over time (e.g., appointment keeping, managing medications). Autistic young adults who participated via proxy-report showed no significant changes in healthcare independence over time, and significant improvement on the management of activities subdomain between baseline and 12 months. Changes in healthcare independence were associated with interactions with providers, individual health changes, consistent support needs, and community resources.

**Conclusions:**

At one autism-specialized primary care clinic, some autistic young adults may demonstrate improvements in healthcare independence, but other autistic young adults may require additional support strategies to increase healthcare independence. Future studies among larger samples are needed to obtain generalizable understanding of healthcare independence for autistic adults.

## Highlights

• Healthcare independence refers to taking charge of one’s healthcare.
• Understanding healthcare independence is important to equitable healthcare design.
• We measured change in autistic young adults’ healthcare independence over time.
• Autistic young adults may require specialized support to increase healthcare independence.

## 1 Introduction

Health management and participation in healthcare are often challenging for autistic adults due to factors such as complex health status,^1^, ^2^ poor patient-provider communication and relationships,^3^, ^4^, ^5^ and systemic inequities in healthcare commonly experienced by autistic people. For example, accommodation of autistic adults’ individual needs is rare in healthcare settings,^6^, ^7^, ^8^ despite the legal mandate for providers to make reasonable accommodations for individuals with disabilities.^9^ These factors can make management of health conditions more difficult for autistic adults and may contribute to autistic adults’ higher mortality rates than non-autistic adults^10^, ^11^ and dissatisfaction with healthcare.^6^, ^12^, ^13^ Recent national-level initiatives from federal organizations in the United States have emphasized the need to improve healthcare for the broader population of people with disabilities^14^ and autism advocacy groups have published parallel goals about improving healthcare services and health outcomes for autistic people throughout adulthood.^15^

Promoting growth in healthcare independence is one way for providers to help their autistic adult patients take a leading role in their healthcare and is congruent with healthcare quality features like patient-centeredness.^16^ Healthcare independence refers to an individual’s ability to take responsibility for their health^17^ by using specific skills like scheduling appointments, communicating with providers (e.g., describing symptoms), and adhering to treatments or therapies (e.g., taking medications as prescribed). Some evidence suggests that autistic people have lower healthcare independence than other clinical populations,^18^ but more research in this area is needed, particularly among the growing population of autistic adults. For some autistic adults, such as those with high support needs or a legal guardian, supporters often play a primary role in healthcare management.^19^ For these individuals, healthcare independence may be limited. However, for many autistic adults’ healthcare independence may improve over time when they are provided opportunities to practice healthcare skills, and when their needs as an autistic individual are met in the setting where they receive care.

In our prior work, we documented the effectiveness of a specialized patient-centered primary care clinic for autistic adults at meeting autistic adults’ healthcare needs.^20^ In this mixed methods study, we build upon our previous work by quantifying changes in healthcare independence over time among autistic adult patients at this clinic. Additionally, we interview autistic adults and supporters of autistic adults to understand factors that contextualize observed healthcare independence over time. While our findings are inherently tied to the primary care clinic from which participants were recruited, this work aligns with national goals to improve healthcare for this population.^14^, ^15^

## 2 Methods

### 2.1 Study design

We used an explanatory sequential mixed methods approach, which is a type of mixed methods design with two distinct phases.^21^ Quantitative data are collected in the first phase and are used to inform the subsequent phase of qualitative data collection. Qualitative results are then integrated with quantitative results to help explain and contextualize select quantitative findings.^22^ We collected longitudinal quantitative data in the form of surveys of autistic young adults and supporters of autistic young adults on a validated measure of healthcare independence called the Transition Readiness Assessment Questionnaire (TRAQ).^23^ Participants completed the measure at baseline, six months, and 12 months. At baseline participants provided demographic information and completed questionnaires about executive functioning and repetitive behaviors so that we could characterize the sample and control for the possible associations between these factors and healthcare independence. Qualitative data were obtained from interviews with participants who completed the survey and were used to understand aspects of care that contributed to or detracted from healthcare independence among autistic young adults.

### 2.2 Study setting

This study was conducted at a local primary care clinic in the Midwest United States that is affiliated with a larger university-based hospital system. The clinic specializes in primary care for autistic adults and has been described previously in detail.^24^, ^25^ Briefly, providers and staff have specialized training to care for autistic adults, and the clinic offers pre-visit resources and modifications to healthcare visits to accommodate the needs of individual patients. For example, short videos are available on the clinic website to set expectations for common medical procedures, and providers offer flexibility in scheduling to accommodate patients who may need longer appointment times.

### 2.3 Participants

To be included in the study, autistic young adults had to be 1) a new patient at the clinic, 2) at least 18 years of age, and 3) able to understand study goals and provide informed consent. Supporters of autistic young adults had to be the legal guardian of an autistic young adult (aged 18+ years) who was a new patient at the clinic.

## 3 Quantitative phase

### 3.1 Recruitment procedures and data collection

We used multiple recruitment avenues for this study. Study information and a survey link was provided to potential participants during a telephone intake appointment with the clinic nurse and via fliers at the clinic. The link directed to the main survey page, which included all information needed for informed consent. We also conducted in-person recruitment where a study team member not affiliated with the clinic provided study information to potential participants after the autistic young adult’s medical visit; study team members provided a brief verbal description of the study and stated that participation was voluntary, could be stopped at any time, and would not impact the patient’s medical care or relationship with the clinic. Interested participants were given the option of participating using a provided iPad in the clinic or taking flier to complete the survey at their convenience.

Emails with a survey link were sent to participants when they were due for their 6- or 12-month follow-up. If participants did not complete the survey via the email-provided link but had a medical appointment close to their follow-up date, we attended the clinic and offered them a study iPad on which to complete the survey. If they did not have a medical appointment near their follow-up date, we contacted participants via the telephone number that they provided in the baseline survey to complete for follow-up data collection. Participants received a $50 Amazon gift code for completing the baseline survey, and a $25 gift code for completing each follow-up survey. Data were collected between August 2020 and January 2023.

### 3.2 Study measures

#### 3.2.1 Transition Readiness Assessment Questionnaire (TRAQ)

The TRAQ is a self-report measure of healthcare self-management skills and healthcare utilization.^23^ The measure has 20 items that comprise five subdomains, which are: 1) talking to providers, 2) managing medications, 3) appointment keeping, 4) tracking health issues, and 5) managing daily activities. Items are task-focused (e.g., “Do you fill a prescription if you need to?” “Do you tell the doctor or nurse what you are feeling?”) and are rated on a 5-point scale, where higher scores indicate greater independence with the task described in the item. All items are averaged to give a TRAQ total score ranging from 1 to 5, and items within each subdomain are also averaged to produce five subdomain scores that also range from 1 to 5. The TRAQ questionnaire was designed and validated among adolescents and young adults with intellectual and developmental disabilities, including autism, and has shown good internal consistency and criterion validity.^23^, ^26^

The TRAQ questionnaire completed by autistic adults was administered in its original format. Because we recruited autistic young adults and supporters of autistic young adults in this study, we slightly modified the wording of the TRAQ questionnaire for supporters before data collection. For supporters, we changed the wording of the instructions from “Please check the box that best describes your skill level in the following areas that are important for transition to adult health care” to “When answering the following questions, please think about the autistic adult (patient) for whom you provide care. Please check the box that best describes the patient’s skill level in the following areas that are important for transition to adult health care.” Additionally, we changed the wording of questions from second person (e.g., “Do you fill a prescription if you need to?”) to third person (e.g., “Do they fill a prescription if they need to?”). Last, we changed response scale options from first person (e.g., “Yes I always do this when I need to”) to third person (e.g., “Yes, they always do this when they need to”). The TRAQ was administered to all participants at all time points.

#### 3.2.2 The Adult Repetitive Behaviour Questionnaire-2A (RBQ-2A)

The RBQ-2A is a self-report measure that contains 20 items and assesses the frequency of RRBs.^27^ RRBs are defined as: 1) repetitive movements, use of objects, or speech, 2) insistence on sameness, inflexible adherence to routines, or ritualistic behavior, 3) intense or restricted interests, and 4) sensory processing differences.^28^ The RBQ-2A examines RRBs in two domains: 1) repetitive motor behaviors and 2) insistence on sameness. Items within each domain are scored on a scale of 1–3, where higher scores indicate more frequent RRBs. All items are averaged to produce a total RBQ-2A score, and domain scores are averaged to produce domain-specific scores. The RBQ-2A has demonstrated good internal consistency reliability and convergent validity with the Autism-Spectrum Quotient.^29^ In this study, autistic adults who participated via self-report completed the RBQ-2A at baseline.

#### 3.2.3 Repetitive Behavior Scale – Revised (RBS-R)

The RBS-R is a proxy-report measure with 44 items that assesses RRBs in five domains: 1) stereotypic behavior, 2) self-injurious behavior, 3) compulsive behavior, 4) ritualistic/sameness behavior, and 5) restricted interests.^30^, ^31^ Items are scored on a scale ranging from 0 to 3, with higher scores indicating that RRBs create more problems in the individual’s life. A total RBS-R score is produced by averaging all item scores. A score specific to each domain is also obtained by averaging scores for items within each domain. Because the number of items in each domain is different, possible scores within each domain vary. Stereotypic behavior possible scores range from 0 to 27, self-injurious behavior scores range from 0 to 24, compulsive behavior scores range from 0 to 18, ritualistic behavior scores range from 0 to 36, and restricted interests scores range from 0 to 9. In this study, supporters who provided a proxy-report completed the RBS-R at baseline.

#### 3.2.4 Behavior Rating Inventory of Executive Function – Adult Version (BRIEF-A)

The BRIEF-A is a measure of executive functioning skills with 75 items that comprise two indexes. The first index is a behavioral regulation index, (BRI) which measures ability to regulate behavior and emotional responses. The BRI is comprised of four subscales: 1) inhibit, which assesses inhibitory control and impulsivity, 2) shift, which assesses the ability to transition easily between different situations or activities as needed, 3) emotional control, which assesses the ability to modulate emotional responses, and 4) self-monitor, which assesses social and interpersonal awareness. The second index is a metacognition (MC) index, which measures the respondent’s ability to initiate activities, generate problem-solving ideas, sustain working memory, plan and organize problem-solving approaches, monitor success and failure in problem solving, and organize their environment. The MC index is comprised of five subscales: 1) initiate, which measures ability to start a task or activity and independently generate ideas or problem-solving strategies, 2) working memory, which measures ability to hold information in the mind to complete a task, 3) plan/organize, which measures ability to manage current and future task demands, 4) task monitor, which measures ability to monitor one’s own successes and failures and correct mistakes, and 5) organization of materials, which measures ability to organize work, living, and storage spaces. Items are rated on a three-point scale, with higher scores indicating greater difficulty with executive functioning. The BRIEF-A is available in both a self-report and proxy-report version. Both versions have demonstrated good internal consistency and test-retest reliability, and convergent validity with other measures of executive function. The measure has been validated for use among adults with a range of developmental and neurologic conditions.^32^

### 3.3 Analysis

We used descriptive statistics to describe participant characteristics and scores on the surveys. Categorical variables are reported as frequencies and percentages, and continuous variables are reported as means and standard deviations. To measure change in healthcare independence over time, we used repeated measures generalized linear mixed models, run separately for autistic young adults who participated via self-report and supporters who provided proxy reports. The primary dependent variable was TRAQ total score, with individual TRAQ domains as secondary dependent variables. The primary independent variable was time. All models controlled for the autistic adults’ age, gender, race/ethnicity, repetitive behaviors, executive functioning, and number of clinic visits.

## 4 Qualitative phase

### 4.1 Recruitment procedures and data collection

All participants who completed the 12-month follow-up survey were contacted via email about participation in a phone-based interview. All interviews were conducted by BH, were approximately 20–40 min in length, were audio recorded, and later transcribed via a third-party transcription service. Interviews were semi-structured and followed an interview guide based on the consolidated framework for implementation research (CFIR).^33^ Interview guides for autistic young adults and supporters were the same except for modification of phrasing of questions from first-person for autistic adults to-third person for supporters. Aligning with the CFIR, the interview guides included questions pertaining to individual characteristics (e.g., “Can you tell me about your most recent experience at CAST?”), intervention characteristics (e.g., “What kinds of changes or alterations do you think need to be made to improve your experience at CAST?), and characteristics of the setting/environment (e.g., ”How does the environment of the clinic (size, physical layout, sensory features) affect the care you receive through CAST?). To integrate the quantitative and qualitative datasets, some additional questions were asked to help explain select quantitative findings (e.g., “Do you think there has been a change in your skills at completing healthcare tasks like taking medications?’”). Participants were emailed a $50 Amazon gift code for participating in an interview. Data were collected between January and December of 2022.

### 4.2 Analysis

The codebook was initially comprised of four codes based on our theoretic framework of the CFIR 1) CAST characteristics, 2) patient characteristics, 3) personnel characteristics, and 4) setting and environment.^33^ First, four team members (DR, AC, DG, BH) (called coders) independently coded one transcript to pilot test the codebook, and then all team members met to identify needed modifications to the code descriptions. Percent agreement was calculated each time the team met to guide our formative development of the codebook; for example, if we found low agreement (<90%) for a particular code, we revised our codebook to clarify the definition of that code. Each time a change was made to the codebook, coders independently re-coded previously coded transcripts and 1–2 new transcripts. Once changes to the codebook were no longer being made, remaining transcripts were coded independently by two coders to promote trustworthiness.^34^ The final codebook consisted of the following codes 1) CAST systemic characteristics, 2) CAST accommodations to care, 3) CAST personnel characteristics, 4) patient characteristics, 5) community resources or experiences, and 6) recommendations. Coders achieved at least 90% agreement on all codes. All coding was conducted in NViVo. Last, we examined coded data to identify themes of broader significance. We evaluated cross-connections to identify themes that bridged across our initial framework of codes as well as any potential subthemes.

### 4.3 IRB approval

This study was reviewed and approved by the Institutional Review Board at The Ohio State University (2019B0436).

## 5 Results

### 5.1 Participant characteristics

We included n = 27 autistic young adults who participated via self-report and n = 21 supporters of autistic young adults who provided a proxy-report in our quantitative analysis. Of the n = 27 participants who participated via self-report, 74.07% completed the six-month survey, and 62.95% completed the 12-month survey. Of the n = 21 supporters who provided a proxy report, 76.19% completed the six-month survey, and 76.19% completed the 12-month survey. Across both groups, retention at six-months was 75%, and at 12-months was 68.75%. Of these participants, we completed interviews with n = 6 autistic young adults and n = 5 supporters of autistic young adults. Demographic characteristics of autistic young adults who participated in the survey and/or interviews, as well as demographic information of supporters, are provided in Table 1. Baseline scores on the RBQ-2A, RBS-R, and BRIEF-A for all participants are provided in Table 2. Baseline TRAQ total scores and subdomain scores, as well as 6-month and 12-month scores, are provided in Table 3.

**Table 1:** Participant demographic characteristics.

	Survey Participants				Interview Participants		
	Autistic adults (self-report) n = 27	Autistic adults (proxy-report)^a^ n = 21	Supporters n = 21		Autistic adults (self-report) n = 6	Autistic adults (proxy-report)^a^ n = 5	Supporters n = 5
**Gender** n(%)							
Female	6 (22.22)	5 (23.81)	19 (90.48)		2 (33.33)	1 (20.00)	5 (100.00)
Male	18 (66.67)	16 (76.19)	2 (9.52)		3 (50.00)	4 (80.00)	0 (0.00)
Non-binary	3 (11.11)	0 (0.00)			1 (16.67)	0 (0.00)	0 (0.00)
**Age** M(SD)	20.30 (2.03)	20.14 (1.49)	50.53 (7.46)		19.50 (1.87)	20.60 (1.14)	49.00 (5.48)
**Race** n(%)							
Asian,>1 race, other race, Hispanic	1 (3.70)	4 (19.05)	2 (9.52)		0 (0.00)	0 (0.00)	0 (0.00)
Black	3 (11.11)	4 (19.05)	4 (19.05)		1 (16.67)	1 (20.00)	1 (20.00)
White	23 (85.19)	13 (61.90)	15 (71.43)		5 (83.33)	4 (80.00)	4 (80.00)
**Highest level of education** n(%)							
High school diploma or less	16 (59.26)	21(100.00)	5 (23.81)		2 (33.33)	5 (100.00)	0 (0.00)
Some college or more	11 (40.74)	0 (0.00)	16 (76.19)		4 (66.67)	0 (0.00)	5 (100.00)
**Annual household income** n(%)							
<$50,000	8 (29.63)	–	5 (23.81)		2 (33.33)	–	0 (0.00)
> $50,000	6 (22.22)	–	14 (66.67)		1 (16.67)	–	4 (80.00)
Not reported	13 (48.15)	–	2 (9.52)		3 (50.00)	–	1 (20.00)
**Marital status** n(%)							
Single or divorced	26 (96.30)	–	6 (28.57)		5 (83.33)	–	0 (0.00)
Married or partnered	1 (3.70)	–	15 (71.43)		1 (16.67)	–	5 (100.00)
**Employment** n(%)		–					
Not employed or retired	17 (62.96)	–	3 (14.29)		5 (83.33)	–	0 (0.00)
Employed full time or part time	10 (37.04)	–	18 (85.71)		1 (16.67)	–	5 (100.00)

a Demographic characteristics for the autistic adults for whom supporters provided proxy reports; M(SD) = Mean (Standard Deviation).

**Table 2:** Self- and proxy-reports of repetitive behaviors and executive functioning.

	Autistic adults who self-reported n = 27	Autistic adults with proxy reports n = 21
**RBQ-2A** M(SE)	1.8 (0.1)	–
Repetitive motor behaviors	2.0 (0.1)	–
Insistence on sameness	1.9 (0.1)	–
**RBS-R** M(SE)	–	51.6 (6.0)
Stereotypic behavior	–	8.5 (1.3)
Self-injurious behavior	–	3.9 (0.9)
Compulsive behavior	–	3.8 (0.6)
Ritualistic behavior	–	10.9 (2.1)
Restricted interests	–	4.6 (0.7)
**BRIEF-BRI** M(SE)	57.3 (2.9)	60.4 (3.6)
Inhibit	14.2 (0.8)	15.2 (0.9)
Shift	12.4 (0.7)	12.6 (0.8)
Emotional control	19.3 (1.2)	20.2 (1.5)
Self-monitor	11.4 (0.7)	12.4 (0.9)
**BRIEF-MC** M(SE)	76.6 (3.8)	78.2 (5.7)
Initiate	15.7 (0.8)	15.6 (1.1)
Working memory	15.7 (0.9)	17.2 (1.1)
Plan/organize	18.6 (1.0)	19.3 (1.6)
Task monitor	11.4 (0.5)	11.9 (1.0)
Organization of materials	15.0 (0.9)	15.2 (1.3)

M(SE) = Mean(Standard Error); RBQ-2A = Repetitive Behavior Questionnaire-2A; RBS-R = Repetitive Behaviors Scale-Revised; BRIEF-BRI = Behavior Rating Inventory of Executive Function-Behavioral Regulation Index; BRIEF-MC = Behavior Rating Inventory of Executive Function-Metacognition Index.

**Table 3:** Mean and standard errors of TRAQ scores over time.

	*Autistic adults who self-reported			**Autistic adults with proxy-reports		
	Baseline N = 27	6 months N = 20	12 months N = 17	Baseline N = 21	6 months N = 16	12 months N = 12
**TRAQ-Total**	3.3 (0.3)	3.3 (0.3)	3.7 (0.3)^ab^	1.5 (0.2)	1.7 (0.2)	1.7 (0.2)
Appointment Keeping	2.9 (0.4)	2.6 (0.5)	3.2 (0.4)^b^	1.2 (0.1)	1.1 (0.1)	1.2 (0.1)
Managing Medications	3.2 (0.4)	3.8 (0.4)^a^	3.7 (0.4)^a^	1.3 (0.2)	1.4 (0.2)	1.3 (0.2)
Talking to Providers	4.6 (0.2)	4.5 (0.2)	4.6 (0.2)	2.2 (0.5)	2.3 (0.5)	2.6 (0.5)
Tracking Health Issues	3.2 (0.4)	3.1 (0.4)	3.9 (0.4)^ab^	1.1 (0.2)	1.5 (0.2)	1.5 (0.2)
Management of Activities	3.9 (0.3)	3.7 (0.3)	4.1 (0.3)	2.5 (0.4)	3.1 (0.4)	3.2 (0.4)^a^
***Cronbach’s alpha coefficient	α = .80	α = .78	α = .81	α = .82	α = 0.78	α = 0.74

M(SE) = Mean(Standard Error)

*Controlling for age, gender, number of visits, race/ethnicity, RBQ-2A, BRIEF-BRI, BRIEF-MC

**Controlling for age, gender, number of visits, race/ethnicity, RBS-R, BRIEF-BRI, BRIEF-MC; note that this is a distinct sample of autistic adults from those who participated via self-report

***Alphas should be interpreted cautiously considering sample size

^a^Significantly different from baseline (p < 0.05)

^b^Significantly different from 6-months (p < 0.05).

### 5.2 Healthcare independence over time

**Fig. 1:**
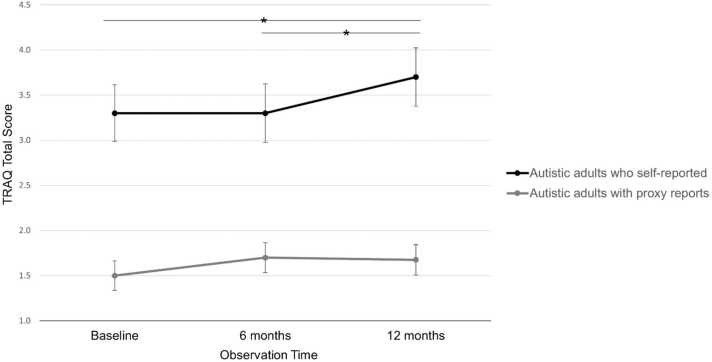
Mean TRAQ total scores *Significant improvement among self-report participants, p < 0.01; Error bars represent standard error.

Our multivariable analysis controlling for sex, race/ethnicity, age, restrictive/repetitive behaviors, executive functioning, and number of clinic visits revealed autistic young adults who self-reported demonstrated significant increases in TRAQ total scores from baseline to 12 months (p = .01) and from 6 months to 12 months (p = .01) (Fig. 1). Autistic young adults who participated via self-report also showed some significant improvements in TRAQ subdomain scores over time (Table 3). Tracking health issues improved from 6 months to 12 months (p < 0.01) and from baseline to 12 months (p = .001). Appointment keeping improved from 6 months to 12 months (p = .02). Managing medications improved from baseline to 6 months (p = .04) and from baseline to 12 months (p = .03).

Among autistic young adults who participated via proxy-report, TRAQ total scores and scores on most subdomains increased slightly over time (Table 3). However, there were no significant changes in TRAQ-total over time, and few significant changes in subdomain scores. The one subdomain for which scores did improve over time was management of activities, where scores significantly increased from baseline to 12 months (p = .04).

### 5.3 Factors contextualizing observed trends in healthcare independence over time

We identified four over-arching themes that contextualize observed trends in autistic young adults’ healthcare independence over time: (1) patient-provider interactions foster healthcare independence; (2) health and life changes affect healthcare independence; (3) consistency in support needs over time; and (4) community resources that increase healthcare independence.

#### 5.3.1 Patient-provider interactions foster healthcare independence

Some autistic young adults shared how interacting with providers increased their healthcare independence. For example, one autistic young adult said “I've become a lot more comfortable because I know that I have a provider that will listen to me and won't just talk over me to my parents because that was my experiences a lot growing up. It was like they were talking about me, but not to me.” Similarly, another autistic young adult said initiating care at CAST was their “first step into healthcare independence.” Autistic young adults and supporters appreciated that CAST providers spoke directly to and listened to autistic young adults. One autistic young adult described how this helped them feel more involved in their care:

“[My provider] always really made an effort to involve me in my own care […] A lot of times providers will see autism or any other developmental disability, they'll see that on a chart and assume that you're not able to understand or have that conversation with them. And [my provider] definitely, [is] not like that. [My provider] never talked down to me or acted like I didn't know what I was talking about […].”

– Autistic adult

Autistic young adults said interacting with their provider helped them “become a lot more competent in asking for what I need and advocating for myself.” They described how CAST approached their care as more of a “dialog” than other places. One supporter described how the providers speaking directly to the autistic young adult for whom they provided care helped them to develop responsibility, stating “[The provider] helped [patient name] voice his opinions and take steps toward being responsible for his own care.”

#### 5.3.2 Health and life changes affect healthcare independence

For some autistic young adults, changes in healthcare independence were spurred by new health challenges or life changes. One autistic young adult described how their independence in managing medications decreased as their regimen increased in complexity. However, this same individual shared that after attending a nutrition program, they found out it was not covered by their insurance; this created an opportunity for them to learn how to check their insurance coverage before using a service going forward. Another autistic young adults’ healthcare independence increased when life circumstances changed, saying, “I've been forced to make my own appointments and go to them by myself and be in charge of prescriptions since I was in my early teens, which wasn't necessarily my choice, but I understand with my family's situation how that had to happen.”

#### 5.3.3 Consistency in support needs over time

Supporters frequently described that the autistic young adults for whom they provide care have some healthcare skills, but that it was important that they had an advocate because they had high support needs (e.g., limited use of verbal language). For example, one supporter said:

“[…] my daughter, she could never independently go to a doctor and communicate her needs and there's really no way to bridge that gap because her whole life, there'll always be either a parent or a guardian or somebody that knows her really well that would go with her.”

– Supporter

Another supporter shared that their son’s capacity to answer questions from providers was limited, but he was often able to answer when asked “direct questions.” Many supporters expected these support needs to be consistent over time. One supporter recommended trainings to help autistic young adults with higher support needs learn new healthcare skills, saying “If just there was some sort of a system where [autistic young adults] were taught how to maintain appointments, how to make appointments and things like that […] if CAST had a program like that, I'm sure that people would attend.”

#### 5.3.4 Community resources that increase healthcare independence

Community resources, independent of CAST, helped some autistic young adults learn new skills. One supporter said their son’s participation in a community educational services program increased his skills in answering questions and making a calendar of appointments: “He was told what he had to do and then the teachers and staff were like, "This is your responsibility. You're an adult," and he did learn how to do it. So, yeah. I do think that's what fed his growth in those areas.” Another supporter said that their daughter’s job-skills program helped her to improve her communication skills, which ultimately translated to increased communication with her healthcare provider.

## 6 Discussion

Autistic adults often experience declines in healthcare service use and discontinuities in care^35^, ^36^ upon transitioning to adult healthcare systems, leading to high unmet healthcare needs.^37^ Thus, it is important to support autistic people before, during, and after transitioning to adult healthcare systems. By characterizing healthcare independence over time among autistic young adults who established care at a specialized patient-centered primary care clinic, our study addresses the nationally-recognized need to develop and evaluate improved models of healthcare for autistic people.^15^ Quantitative data revealed autistic adults who participated via self-report showed significant increases in healthcare independence over 12 months, while those who participated via proxy-report did not. Qualitative data revealed that healthcare independence over time was related to patient-provider interactions, health and life changes, consistency in support needs over time, and engagement with community resources.

Autistic young adults who participated via self-report showed significant increases in healthcare independence over time while those for whom we had proxy reports did not. Participants who provided a self- report differed on some demographic characteristics from proxy reporters, which could help explain this finding as people of different demographic characteristics may perceive disabilities differently.^38^ Additionally, differing perceptions of disabilities and health states between disabled people and proxy respondents is a known phenomenon,^39^, ^40^, ^41^ with proxy respondents typically reporting more severe impairments or worse health states than what disabled people report themselves. This may contribute to why we did not observe improvement in healthcare skills among individuals who participated via proxy report. Alternatively, this finding may be related to differing healthcare needs and trajectories for health-related outcomes for autistic adults with higher support needs (often due to co-occurring intellectual disability) relative to those with less extensive support needs.^42^, ^43^, ^44^, ^45^ For autistic adults with more extensive support needs, improvements in healthcare independence may improve over longer periods of time than the 12 month window of observation in this study. Autistic adults with lower support needs may be uniquely poised to respond to and benefit from approaches to care that promote increasing healthcare independence. Autistic adults’ inclusion during patient-provider interactions, for example, was described as promoting healthcare independence mostly by autistic young adults who participated via self-report. Interestingly, however, this group of participants showed no significant improvement over time on the talking to providers TRAQ subdomain. This finding warrants further study, but may be related to the fact that for some autistic adults, communicative abilities often fluctuate depending on factors such as sickness, present sensory demands, or mode of communication.^4^, ^46^, ^47^ As such, interactions with providers may increase autistic adults’ comfort in talking to providers, but other obstacles may prevent application of these skills. Alternatively, that scores on this subdomain did not increase over time may be because they were relatively high at baseline, limiting the degree to which scores could improve over time due to a possible ceiling effect. Relatively high scores at baseline may also help explain why we did not observe significant improvements on the management of activities subscale for autistic young adults who participated via self-report.

Autistic young adults who participated via proxy-report were often described by supporters as having higher support needs that were consistent over time and across contexts. These autistic young adults may not have substantial roles in handling their own healthcare tasks as often, which may explain the absence of significant improvements in their healthcare independence over time for most domains. This group of participants did demonstrate significant improvement on the management of activities TRAQ subdomain, which contains items describing tasks that are largely not healthcare specific (e.g., “Do you help plan or prepare meals/food?”). Autistic young adults with higher support needs may participate in these daily tasks more frequently than healthcare-specific tasks measured in other TRAQ subdomains. It is also possible that the improvements in this domain were predominately attributable to resources outside of CAST, as supporters noted that autistic adults learned skills (e.g., how to keep a calendar of appointments) through community programming. This finding highlights that autistic adults may learn healthcare-relevant skills from community programming, which can play an important role in promoting autistic adults’ healthcare independence, in addition to medical providers. Some supporters suggested that developing programming at CAST to teach healthcare skills would be helpful for the autistic young adult for whom they provided care. This may require integrating effective components of community-based resources (e.g., mentoring)^48^ into healthcare independence programming, or exploring other evidence-based ways to support autistic young adults in fostering increased healthcare independence.

Our study included autistic young adults and supporters from a local primary care clinic that specializes in caring for autistic adults, which may limit the generalizability of our findings beyond CAST. However, our findings are a valuable initial contribution toward understanding healthcare independence over time among autistic adults when they receive patient-centered care from knowledgeable providers. Our use of mixed methods allowed us to qualitatively explore factors that provide context for the observed trends in healthcare independence over time, but we cannot test or infer a causal relationship between these factors and changes in healthcare independence, and we were not able to control for all autism diagnostic criteria in our analyses. Data were collected over 12 months, and changes in healthcare independence may be different for autistic young adults over longer periods of time. Additionally, there was some attrition across measurement periods, and our study did not include a control group of non-CAST patients so we could not determine the extent to which any significant changes in healthcare independence over time were attributable to natural developmental changes. Our sample of autistic young adults was relatively homogenous in terms of gender and racial identities, and findings may not generalize to autistic adults with different gender or racial identities.

Also, while the TRAQ was developed and validated using a sample that included autistic people and people with other complex healthcare needs,^26^ the psychometric properties of this questionnaire among samples consisting exclusively of autistic adults have not been established. Additionally, our modified version of the TRAQ for family members has not been psychometrically evaluated; due to our limited sample size, psychometric evaluation of this modified questionnaire was not possible in this study. Since we initiated data collection for this study, a new version of the TRAQ and a tool to measure health-related independence specifically for autistic people were published.^49^, ^50^ In future work, these updated measures should be used to capture the most valid data on healthcare independence.

## 7 Conclusion

We used quantitative surveys to measure autistic young adults’ healthcare independence over time during the first year of receiving healthcare at a specialized primary care clinic designed with and for autistic adults. We used qualitative interviews to characterize factors that were associated with healthcare independence over time. Autistic young adults who participated via self-report showed significant improvements in healthcare independence over time, but autistic young adults who participated via proxy report did not. Qualitative data revealed factors related and unrelated to CAST that provide context for observed trends in healthcare independence over time. Our findings suggest that some autistic young adults demonstrate significant improvements in healthcare independence within the first year of receiving healthcare at a specialized primary care clinic, while additional support strategies to foster healthcare independence may need to be developed for other autistic young adults.

## Funding

This work was supported by 10.13039/100000073Autism Speaks (Grant #11761).

## Ethical statement

Informed consent was obtained for experimentation with human subjects. We did not use AI or AI technologies at any point during this project.

## CRediT authorship contribution statement

**Daniel Gilmore**: Investigation, Data curation, Formal analysis, Writing – original draft, Writing – review & editing. **Deondray Radford:** Formal analysis, Writing – review & editing. **Alex Coyne:** Formal analysis, Writing – review & editing. **Christopher Hanks:** Conceptualization, Resources, Writing – review & editing. **Daniel Coury:** Conceptualization, Writing – review & editing. **Amy Hess:** Resources, Writing – review & editing. **Jennifer Garvin:** Conceptualization, Writing – review & editing. **Brittany N. Hand:** Conceptualization, Methodology, Formal analysis, Writing – original draft, Writing – review & editing, Supervision, Funding acquisition.

## Declaration of Competing Interest

The authors declare the following financial interests/personal relationships which may be considered as potential competing interests: Brittany N. Hand reports financial support was provided by Autism Speaks. Christopher Hanks reports a relationship with The Center for Autism Services and Transition that includes: employment. Christopher Hanks is the founder and medical director of CAST.

## Data Availability

Data will be made available on request.

## References

[1] Hand, Angell A.M., Harris L., Carpenter L.A. (2020). Prevalence of physical and mental health conditions in Medicare-enrolled, autistic older adults. Autism.

[2] Lai M.C., Kassee C., Besney R. (2019). Prevalence of co-occurring mental health diagnoses in the autism population: a systematic review and meta-analysis. Lancet Psychiatry.

[3] Cummins C., Pellicano E., Crane L. (2020). Autistic adults’ views of their communication skills and needs. Int J Lang Commun Disord.

[4] Doherty M., Neilson S., O’Sullivan J. (2022). Barriers to healthcare and self-reported adverse outcomes for autistic adults: a cross-sectional study. BMJ Open.

[5] Voillemont C., Imbault E., Schoenberger M., Di Patrizio P. (2022). Care and management of adults with autism spectrum disorder in family practice: difficulties experienced by general practitioners. Fam Pract.

[6] Camm-Crosbie L., Bradley L., Shaw R., Baron-Cohen S., Cassidy S. (2019). People like me don’t get support’: autistic adults’ experiences of support and treatment for mental health difficulties, self-injury and suicidality. Autism.

[7] Lipinski S., Boegl K., Blanke E.S., Suenkel U., Dziobek I. (2022). A blind spot in mental healthcare? Psychotherapists lack education and expertise for the support of adults on the autism spectrum. Autism.

[8] Strömberg M., Liman L., Bang P., Igelström K. (2022). Experiences of sensory overload and communication barriers by autistic adults in health care settings. Autism Adulthood.

[9] Harkin T. Text - S.933 - 101st Congress (1989–1990): Americans with Disabilities Act of 1990; 1990. 〈http://www.congress.gov/〉. Accessed January 18, 2023.

[10] Hwang Y.I. (Jane), Srasuebkul P., Foley K.R., Arnold S., Trollor J.N. (2019). Mortality and cause of death of Australians on the autism spectrum. Autism Res.

[11] Krantz M., Dalmacy D., Bishop L., Hyer J.M., Hand B.N. (2023). Mortality rate and age of death among Medicare-enrolled autistic older adults. Res Autism Spectr Disord.

[12] Anderson C., Butt C. (2018). Young adults on the autism spectrum: the struggle for appropriate services. J Autism Dev Disord.

[13] Nicolaidis (2013). Comparison of healthcare experiences in autistic and non-autistic adults: a cross-sectional online survey facilitatied by an acacemic community partnership. J Gen Intern Med.

[14] US Department of Health and Human Services. People with disabilities — evidence-based resources - healthy people 2030; 2022 [Internet]. 〈https://health.gov/healthypeople/objectives-and-data/browse-objectives/people-disabilities/evidence-based-resources〉. Accessed September 22, 2022.

[15] Interagency Autism Coordinating Committee (IACC). IACC strategic plan for autism spectrum disorder 2018–2019; 2018, p. 77.

[16] Agency for Healthcare Research and Quality. Understanding quality measurement. US Department of Health and Human Services; 2020. 〈http://www.ahrq.gov/patient-safety/quality-resources/tools/chtoolbx/understand/index.html〉. Accessed May 10, 2021.

[17] Heath G., Farre A., Shaw K. (2017). Parenting a child with chronic illness as they transition into adulthood: a systematic review and thematic synthesis of parents’ experiences. Patient Educ Couns.

[18] Beal S.J., Riddle I.K., Kichler J.C. (2016). The associations of chronic condition type and individual characteristics with transition readiness. Acad Pediatr.

[19] Elster N., Parsi K. (2020). Transitioning from adolescence to adulthood with autism spectrum disorder: an overview of planning and legal issues. Psychiatr Clin.

[20] Hand B.N., Coury D.L., Darragh A.R. (2020). Patient and caregiver experiences at a specialized primary care center for autistic adults. J Comp Eff Res.

[21] Ivankova N.V., Creswell J.W., Stick S.L. (2006). Using mixed-methods sequential explanatory design: from theory to practice. Field Methods.

[22] Creswell J.W., Clark V.L.P. (2017).

[23] Wood D.L., Sawicki G.S., Miller M.D. (2014). The Transition Readiness Assessment Questionnaire (TRAQ): its factor structure, reliability, and validity. Acad Pediatr.

[24] Hand B.N., Gilmore D., Harris L. (2021). “They looked at me as a person, not just a diagnosis”: a qualitative study of patient and parent satisfaction with a specialized primary care clinic for autistic adults. Autism Adulthood.

[25] Saqr Y., Braun E., Porter K., Barnette D., Hanks C. (2018). Addressing medical needs of adolescents and adults with autism spectrum disorders in a primary care setting. Autism.

[26] Sawicki G.S., Lukens-Bull K., Yin X. (2011). Measuring the transition readiness of youth with special healthcare needs: validation of the TRAQ—Transition Readiness Assessment Questionnaire. J Pediatr Psychol.

[27] Barrett S.L., Uljarević M., Baker E.K., Richdale A.L., Jones C.R.G., Leekam S.R. (2015). The Adult Repetitive Behaviours Questionnaire-2 (RBQ-2A): a self-report measure of restricted and repetitive behaviours. J Autism Dev Disord.

[28] Turner-Brown L., Frisch M., Vivanti G., Bottema-Beutel K., Turner-Brown L. (2020). Clinical guide to early interventions for children with autism. Best practices in child and adolescent behavioral health care.

[29] Barrett S.L., Uljarević M., Jones C.R.G., Leekam S.R. (2018). Assessing subtypes of restricted and repetitive behaviour using the Adult Repetitive Behaviour Questionnaire-2 in autistic adults. Mol Autism.

[30] Bodfish J.W., Symons F.J., Parker D.E., Lewis M.H. (2000). Varieties of repetitive behavior in autism: comparisons to mental retardation. J Autism Dev Disord.

[31] Lam K.S.L., Aman M.G. (2007). The repetitive behavior scale-revised: independent validation in individuals with autism spectrum disorders. J Autism Dev Disord.

[32] Roth RM, Isquith PK, Gioia GA. Behavior rating inventory of executive function-adult version: professional manual. Psychol Assess Resour; 2005.

[33] Damschroder L.J., Aron D.C., Keith R.E., Kirsh S.R., Alexander J.A., Lowery J.C. (2009). Fostering implementation of health services research findings into practice: a consolidated framework for advancing implementation science. Implement Sci.

[34] Nowell L.S., Norris J.M., White D.E., Moules N.J. (2017). Thematic analysis: striving to meet the trustworthiness criteria. Int J Qual Methods.

[35] Benevides, Carretta H., Graves K. (2017). Health care utilization and costs among transition-age young adult medicare beneficiaries with autism spectrum disorder. Am J Occup Ther.

[36] Vohra R., Madhavan S., Sambamoorthi U., St Peter C. (2014). Access to services, quality of care, and family impact for children with autism, other developmental disabilities, and other mental health conditions. Autism.

[37] Nicolaidis, Raymaker D.M., Ashkenazy E. (2015). Respect the way I need to communicate with you”: healthcare experiences of adults on the autism spectrum. Autism.

[38] Rosenbrock G.J., Mire S.S., Kim H.J., Aguirre-Munoz Z. (2021). Exploring sociodemographic predictors of parents’ perceptions about their children’s autism and their families’ adjustment. Res Dev Disabil.

[39] Mumbardó-Adam C., Andrés-Gárriz C., Sánchez-Pedroche A., Balboni G. (2023). Differences in self and proxy assessments of self-determination in young people with intellectual disability: the role of personal and contextual variables. Behav Sci.

[40] McDonald K.E., Raymaker D.M. (2013). Paradigm shifts in disability and health: toward more ethical public health research. Am J Public Health.

[41] Bamer A.M., McMullen K., Wolf S.E. (2021). Agreement between proxy- and self-report scores on PROMIS health-related quality of life domains in pediatric burn survivors: a National Institute on Disability, Independent Living, and Rehabilitation Research Burn Model System Study. Qual Life Res.

[42] Bishop-Fitzpatrick L., Rubenstein E. (2019). The physical and mental health of middle aged and older adults on the autism spectrum and the impact of intellectual disability. Res Autism Spectr Disord.

[43] Gilmore D., Harris L., Longo A., Hand B.N. (2020). Health status of Medicare-enrolled autistic older adults with and without co-occurring intellectual disability: an analysis of inpatient and institutional outpatient medical claims. Autism.

[44] Hand BN, Benevides TW, Carretta HJ. Suicidal ideation and self-inflicted injury in medicare enrolled autistic adults with and without co-occurring intellectual disability. J Autism Dev Disord; 2019;((Hand B.N., hand.58@osu.edu) School of Health and Rehabilitation Sciences, College of Medicine, The Ohio State University, 453 W 10th Ave, Columbus, OH, United States). DOI: 10.1007/s10803-019-04345-x. 10.1007/s10803-019-04345-x 31858322

[45] Jose C., George-Zwicker P., Bouma A. (2021). The associations between clinical, social, financial factors and unmet needs of autistic adults: results from an observational study. Autism Adulthood.

[46] Harris L., Gilmore D., Hanks C. (2021). “It was surprisingly equivalent to the appointment I had in person”: advantages and disadvantages of synchronous telehealth for delivering primary care for autistic adults. Autism.

[47] Howard P.L., Sedgewick F. (2021). ‘Anything but the phone!’: communication mode preferences in the autism community. Autism.

[48] Cameron C., Townend A. (2021). How might we best support the effective and meaningful employment of autistic people and improve outcomes?. Adv Autism.

[49] Cheak-Zamora N., Petroski G., La Manna A., Beversdorf D., Farmer J. (2021). Validation of the health-related independence for young adults with autism spectrum disorder measure- caregiver version. J Autism Dev Disord.

[50] Johnson K., McBee M., Reiss J., Livingood W., Wood D. (2021). TRAQ changes: improving the measurement of transition readiness by the transition readiness assessment questionnaire. J Pediatr Nurs.

